# Transcatheter Versus Surgical Aortic Valve Replacement in Low-Risk Patients: A Systematic Review of Long-Term Outcomes

**DOI:** 10.7759/cureus.104196

**Published:** 2026-02-24

**Authors:** Karam Khasawneh, Mohamed Alzandani, Ghaeth Khasawneh, John Obeid, Christopher Bobier

**Affiliations:** 1 Medicine, Central Michigan University College of Medicine, Mount Pleasant, USA; 2 Medicine, University of Minnesota, Minneapolis, USA; 3 Health Services Research, Central Michigan University College of Medicine, Mount Pleasant, USA

**Keywords:** aortic stenosis (as), aortic valve replacement, long-term outcomes, low-risk patients, savr, surgical aortic valve replacement, tavr, transcatheter aortic valve replacement

## Abstract

The role of transcatheter aortic valve replacement (TAVR) in patients with aortic stenosis (AS) continues to expand, yet uncertainty remains regarding the long-term outcomes in low-risk populations compared with surgical aortic valve replacement (SAVR). This systematic review examined long-term mortality, stroke, postoperative complications, and valve durability across randomized and observational studies. Randomized data showed similar long-term survival and stroke rates between TAVR and SAVR, while observational studies suggested worse late outcomes following TAVR. TAVR was consistently associated with fewer postoperative complications, whereas durability and re-intervention findings varied across studies. Overall, these results suggest that while TAVR offers meaningful advantages, treatment decisions should be individualized to balance a patient's comorbidities with long-term procedural outcomes.

## Introduction and background

Aortic valve replacement (AVR) is considered the definitive treatment for patients experiencing symptomatic aortic stenosis (AS). Traditionally, surgical aortic valve replacement (SAVR) has been considered the gold standard for patients requiring valve intervention. However, the emergence of transcatheter aortic valve replacement (TAVR) has revolutionized the treatment landscape, particularly for high-risk surgical candidates. Previous research has shown that TAVR provides comparable, and in some cases superior, short-term and long-term outcomes when compared to SAVR in elderly and high-risk patients. For example, one study reported that among 1,414 patients who underwent AVR as a result of AS, patients who received TAVR had a mortality rate of 2.6% compared to 6.2% of patients who received SAVR one year after intervention [1]. Furthermore, 2.9% of patients who received TAVR experienced a stroke within one year of intervention compared to 4.7% of patients who received SAVR [1]. As a result, TAVR use in patients at high risk for normal surgical intervention has steadily increased over time. 

In contrast, the use of TAVR in low-risk patients remains less clear, as the long-term outcomes of TAVR vs SAVR in this cohort have not been thoroughly evaluated. Previous research shows favorable short-term outcomes for TAVR in low-risk populations, but there is limited evidence regarding the long-term effects [2]. Some of these long-term effects include death from any cause, stroke, and postoperative morbidities. In this study, we examine low-risk populations with AS undergoing TAVR and compare them to low-risk patients who underwent SAVR to assess long-term mortality, stroke, postoperative complications, and valve durability. By analyzing these long-term outcomes, this study aims to develop a clearer understanding of the benefits and risks associated with each treatment modality in low-risk populations, further highlighting the role of each TAVR and SAVR in broader patient populations.

## Review

Materials and methods

No institutional review board approval was required as the results were gathered from published data. All authors had full access to the data in the study and took responsibility for its integrity and data analysis.

Search Strategy and Study Selection Criteria

**Figure 1:**
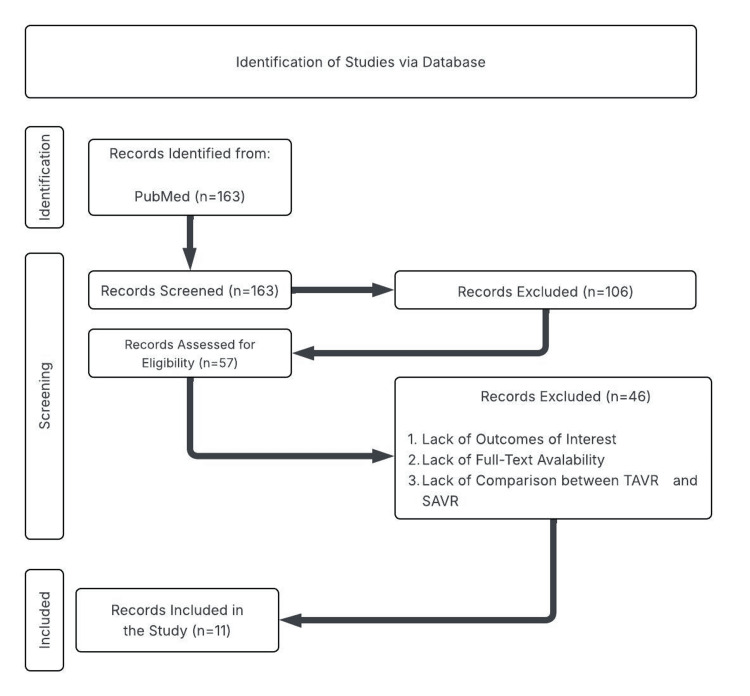
Flowchart for study selection Study selection process according to PRISMA guidelines, detailing database identification, screening, eligibility assessment, and final inclusion of studies [3-13] comparing TAVR and SAVR. PRISMA: Preferred Reporting Items for Systematic Reviews and Meta-Analyses; TAVR: transcatheter aortic valve replacement; SAVR: surgical aortic valve replacement

Our search strategy involved combining key terms and subject headings related to our topic. Database searches were performed using PubMed. Keywords used in our systematic search include "TAVR", "SAVR", "Low-risk", "Stroke", "Mortality", and "post-operative complications". A total of 163 articles from 2017 to 2025 were selected for review based on our search results. After obtaining the results, each author was assigned a certain number of articles to screen for inclusion and exclusion criteria. Our inclusion criteria included peer-reviewed studies, TAVR vs SAVR recipients, and the presence of a discussion about any long-term outcomes of interest in low-risk populations. Low-risk patients were defined according to study-specific criteria, most commonly an STS-PROM (Society of Thoracic Surgeons Predicted Risk of Mortality) score below 4%, preserved functional status, and absence of major surgical contraindications rather than age alone. Long-term outcomes of interest include all-cause mortality, stroke, postoperative complications, and valve durability. Exclusion criteria included studies that only reported on high-risk populations with no discussion of low-risk populations, studies with no discussion of long-term outcomes, and studies that lacked comparison between TAVR and SAVR. Using this method, a total of 57 articles were obtained. Full-text review of each article by every co-author was then performed to ensure the article's relevance to our study. After review and discussion with every co-author, an additional 46 articles were excluded due to the lack of outcomes of interest, the unavailability of full texts, the lack of comparison between TAVR and SAVR, or the duplication of articles. Figure 1 illustrates the flowchart of our search results, highlighting articles that were included/excluded following screening. Using this method, a total of 11 articles [3-13] ranging from the years 2017 to 2025 were selected.

Data Extraction and Statistics 

Each study was assessed for mortality, stroke, postoperative complications, and valve durability among TAVR and SAVR recipients. Outcomes were reported using study-level data as presented in the original publications, including event counts and percentages when available. Studies that reported outcomes pooled across multiple cardiac interventions for AS, without providing data specific to TAVR or SAVR (e.g., studies evaluating medical therapy rather than AVR), were excluded from data reporting.** **Using this method, long-term outcomes of interest were summarized descriptively using study-specific event rates rather than pooled estimates. Furthermore, if some studies reported a long-term outcome of interest for only TAVR or SAVR, but not the other, that data was not reported to avoid under- or overrepresenting a certain outcome of interest for a specific group.

Results

Study Characteristics

The included studies included randomized controlled trials (RCTs), observational cohorts, registry-based analyses, and pooled meta-analyses. The studies were conducted across multinational clinical and administrative settings. The study periods and follow-up varied, ranging from in-hospital outcomes to long-term assessment, reflecting differences in study design and data sources. Table 1 illustrates the characteristics of each of the included studies [3-13].

**Table 1:** Study characteristics We assessed the characteristics of each of the included studies [3-13]. Each study was assessed for setting, study design, study period, and key outcomes. RCTs: randomized controlled trials; AKI: acute kidney injury; TAVR: transcatheter aortic valve replacement; PSM: propensity score-matched

Study ID	Country/setting	Study design	Study period/enrollment timeframe	Key outcomes reported
Grubb et al., 2024 [3]	Multinational; trial-based	Analysis of RCTs	Included studies between the years 2011 and 2019	Re-intervention, valve durability, all-cause mortality
Talanas et al., 2024 [4]	Multinational; pooled trial data	Meta-analysis of RCTs	Included studies published up until 2023	Long-term mortality, stroke
Jørgensen et al., 2021 [5]	Denmark; hospital-based randomized trial	RCT	Enrolled patients between 2009 and 2013 and followed up with them in 5 and 8 years	Mortality, stroke, myocardial infarction, valve durability
Alabbadi et al., 2025 [6]	United States; registry-based	Retrospective observational cohort	Included patients who were treated between the years 2012 and 2019	Long-term mortality, stroke
Auer et al., 2024 [7]	Austria; registry-based	Prospective observational cohort	Included patients who were enrolled between the years 2010 and 2019 with variation in patient follow-up	Mid-term mortality and survival
Gad et al., 2022 [8]	United States; hospital database	Retrospective observational cohort	Included patients who were treated between the years 2012 and 2017	Lower AKI and atrial fibrillation with TAVR
Kramer et al., 2023 [9]	Multinational; hospital-based	Retrospective observational cohort	Not specified	Mortality, perioperative outcomes
Kundu et al., 2020 [10]	Multinational; pooled trial data	Meta-analysis of RCTs	Included studies published up until 2019	Mortality, stroke, cardiovascular outcomes
Kolte et al., 2019 [11]	United States; administrative database	Meta-analysis of RCTs	Included patients who were treated between the years 2012 and 2016	In-hospital mortality, stroke, perioperative complications
Khan et al., 2017 [12]	Multinational; pooled studies	Systematic review and meta-analysis	Included studies published up until 2016	Mortality, stroke
Rawasia et al., 2020 [13]	Multinational; pooled data	Meta-analysis of RCTs and PSM studies	Included studies published up until 2019	Mortality, stroke, perioperative complications

Bias/Quality Assessment

We assessed the risk of bias by using the Newcastle-Ottawa Scale (NOS) [14]. RCTs were not evaluated using NOS and were instead assessed qualitatively. The results of the risk of bias assessment are summarized in Table 2.

**Table 2:** Risk and quality assessment of bias Observational cohort studies were assessed using NOS [14], which evaluated study quality across three domains, selection (0-4 points), comparability (0-2 points), and outcome assessment (0-3 points), for a total possible score of 9. Higher scores indicate lower risk of bias. RCTs and secondary analyses (systematic reviews and pooled analyses) were not assessed using NOS and were instead evaluated qualitatively. RCTs: randomized controlled trials; SVD: structural valve deterioration; BVF: bioprosthetic valve failure; NOS: Newcastle-Ottawa Scale; TAVR: transcatheter aortic valve replacement; SAVR: surgical aortic valve replacement; PSM: propensity score-matched; AKI: acute kidney injury

Study ID	Study design	Quality/risk of bias score	Key bias considerations
Grubb et al., 2024 [3]	Analysis of RCTs	Not applicable	Strong internal validity; durability endpoints influenced by evolving definitions of SVD/BVF and imaging surveillance practices; re-intervention thresholds may vary across centers. NOS not applicable
Talanas et al., 2024 [4]	Meta-analysis of RCTs	Not applicable	Randomization minimizes confounding; long-term follow-up may introduce attrition bias and crossover effects; outcome ascertainment standardized. NOS not applicable to randomized design
Jørgensen et al., 2021 [5]	RCT	Not applicable	Robust randomized design with balanced baseline characteristics; long-term follow-up increases risk of missing data; generalizability may be limited by early-generation valve technology and trial-era selection criteria. NOS not applicable
Alabbadi et al., 2025 [6]	Retrospective observational cohort	6/9	NOS: good cohort selection; limited comparability due to potential selection bias (younger TAVR patients may differ clinically from SAVR); outcome assessment less standardized than RCTs; residual confounding likely
Auer et al., 2024 [7]	Prospective observational cohort	7/9	NOS: strong selection and improved comparability through matching; residual confounding remains (frailty, anatomy, access route); possible time-period and learning-curve bias
Gad et al., 2022 [8]	Retrospective observational cohort	8/9	NOS: retrospective, administrative database design introduces potential for residual confounding despite propensity score matching. Outcomes limited to in-hospital and short-term follow-up without clinical data
Kramer et al., 2023 [9]	Retrospective observational cohort	5/9	NOS: large, representative cohort; limited comparability due to coding-based confounders; outcome assessment dependent on administrative coding; lack of procedural and valve-specific detail
Kundu et al., 2020 [10]	Meta-analysis of RCTs	Not applicable	Quality dependent on included studies; subject to heterogeneity and publication bias; used to contextualize perioperative outcomes rather than for primary risk estimation
Kolte et al., 2019 [11]	Meta-analysis of RCTs	Not applicable	Useful for supporting complication patterns; limited by heterogeneity and possible cohort overlap; not designed for patient-level confounding control
Khan et al., 2017 [12]	Systematic review and meta-analysis	Not applicable	Supports perioperative complication trends; limited by study mix and variability in outcome definitions; potential overlap of cohorts across source datasets
Rawasia et al., 2020 [13]	Meta-analysis of RCTs and PSM studies	Not applicable	Provides consolidated short-term safety signals; heterogeneity in endpoint definitions (AKI, bleeding, vascular complications); not designed to address long-term confounding

All-Cause Mortality

Long-term mortality results differed across study designs and patient age. In one randomized study with a mean follow-up of 5.7 years, there was no significant difference in all-cause mortality between the treatment groups (HR 1.03; 95% CI 0.95-1.11; p=0.54) [4]. Similarly, in low-risk patients enrolled in the Nordic Aortic Valve Intervention (NOTION) trial with eight years of follow-up, mortality rates were comparable between the two groups (51.8% for TAVR vs 52.6% for SAVR; p=0.90) [5].** **

In contrast, certain observational analyses suggested higher long-term mortality following TAVR. For example, among patients <65 years of age with a six-year follow-up, mortality was higher among TAVR recipients compared with SAVR (23.3% vs 10.5%; HR 2.27; 95% CI 1.82-2.83; p<0.001) [6]. Furthermore, in a propensity score-matched (PSM) cohort aged 65-75 years, increased mortality beyond one year was reported in the TAVR group (HR 2.455; 95% CI 2.209-2.728; p<0.001) [7].

Early mortality outcomes were generally favorable or similar for TAVR compared with SAVR. In one cohort with patients <65 years of age, 30-day mortality was similar (1% TAVR vs 1.5% SAVR; p=0.33) [6]. Collectively, randomized long-term evidence demonstrated comparable survival between modalities, whereas some younger and age-stratified observational cohorts reported higher late mortality following TAVR [4-7].

Stroke

Across studies reporting cerebrovascular outcomes, stroke rates were largely comparable between modalities over long-term follow-up. Long-term randomized trial evidence reported no significant difference in any stroke (HR 0.96; 95% CI 0.81-1.13; p=0.41) [4]. In the NOTION trial at eight years, stroke incidence was similar (8.3% TAVR vs 9.1% SAVR; p=0.90) [5]. Among patients <65 years of age with a six-year follow-up, long-term stroke rates remained comparable (2.5% vs 1.7%; HR 1.30; 95% CI 0.74-2.29; p=0.36) [6].

In a hospital database cohort with patients <60 years of age, cerebrovascular accidents occurred less frequently following TAVR (1.9% TAVR vs 3.3% SAVR) [8]. Overall, long-term evidence did not demonstrate a consistent stroke disadvantage for TAVR compared with SAVR [4-6,8].

Postoperative Complications

Multiple studies reported lower postoperative morbidity with TAVR [10-12]. For example, reduced acute kidney injury (AKI) and new-onset atrial fibrillation were reported in TAVR recipients (AKI RR 0.45 and new-onset atrial fibrillation RR 0.21; p<0.001) [13]. Furthermore, hospital cohorts with patients <60 years of age showed lower complications following TAVR vs SAVR, including lower cardiogenic shock (5.5% vs 8.8%), lower aortic dissection (3.2% vs 9.3%), lower AKI (13% vs 21.3%), and reduced need for circulatory support (4.5% vs 34.6%), with all comparisons being statistically significant (p<0.001) [8]. Finally, SAVR was associated with increased prolonged mechanical ventilation and a greater postoperative red blood cell (RBC) transfusion requirement [9].

Valve Durability and Re-intervention Outcomes

Durability-related outcomes were mixed. In the NOTION long-term follow-up, structural valve deterioration occurred less frequently after TAVR (13.9% vs 28.3%; HR 0.42; p=0.0017) [5], while severe bioprosthetic valve failure was similar (8.7% vs 10.5%; p=0.61) [5]. In RCT patients at five years, re-intervention rates were slightly higher following TAVR (2.2% vs 1.5%; HR 1.95; p=0.017) [3], and re-interventions after TAVR tended to occur earlier and were most commonly performed percutaneously, whereas SAVR re-interventions occurred later and were more often surgical [3].

Discussion

All-Cause Mortality

The mortality findings suggest that long-term survival after valve replacement is influenced more by patient selection and long-term factors than by the procedure itself. Randomized trials, which balance baseline risk and anatomy, show similar survival between TAVR and SAVR, indicating that TAVR can achieve durable outcomes when used in appropriately selected low-risk patients [4,5]. In contrast, higher late mortality seen in patients <65 years of age in the observational cohorts likely reflects differences in underlying health status that are difficult to fully adjust for in real-world data, even with propensity matching [6,7]. Patients <65 years of age selected for TAVR may have unmeasured comorbidities, anatomic challenges, or contraindications to surgery that increase long-term risk independent of valve type. These findings suggest that the observed mortality differences in observational studies should not be interpreted as evidence that TAVR is inferior, but rather as a reminder that results may not apply uniformly to all patients, particularly those with complex clinical profiles.

Stroke

Stroke outcomes across the included studies suggest that TAVR and SAVR have similar stroke risk, especially with long-term follow-up [4-6]. This is important because earlier concerns about higher stroke risk with catheter-based procedures were once considered a major limitation of TAVR [15]. Some cohorts with patients <60 years of age showed lower early stroke rates with TAVR [8], which may be related to procedural differences such as avoiding cardiopulmonary bypass and reduced surgical stress. However, these findings should be interpreted cautiously, as differences in patient selection and baseline risk may affect early stroke rates in observational studies. Overall, stroke risk alone should not be a primary factor when choosing between TAVR and SAVR, as results suggest that the outcomes are similar.

Postoperative Complications

Postoperative complications were generally lower with TAVR, which fits with how the procedure is performed. Avoiding open surgery and cardiopulmonary bypass likely helps explain the lower rates of AKI and new-onset atrial fibrillation, complications that often lead to longer hospital stays and worse recovery after SAVR [8,13]. In cohorts with patients <60 years of age, TAVR was also linked to fewer serious complications, including less need for circulatory support and fewer cardiogenic events, suggesting a real short-term recovery advantage in select patients [8]. From a clinical standpoint, these findings support the use of TAVR when faster recovery and avoidance of early complications are important.

Valve Durability and Re-intervention Outcomes

Durability and the need for repeat procedures remain the biggest unanswered issues when choosing between TAVR and SAVR. Long-term data from the NOTION trial suggest that TAVR valves may hold up well over time [5], but other randomized follow-up studies show that repeat interventions can happen earlier after TAVR and for different reasons than after SAVR [3]. This highlights that valve durability is not only about how long the valve leaflets last but also about problems such as paravalvular leak, how the valve interacts with the surrounding tissue, and valve-related infections. The timing and type of repeat procedures also matter, as earlier re-interventions after TAVR may be acceptable for some patients, while later surgical re-interventions after SAVR may carry a higher risk as patients age and develop more comorbidities [3]. Overall, these findings stress the importance of planning treatment with a long-term view, where the initial valve choice takes future interventions into account.

Clinical Implications

From a clinical standpoint, the evidence supports TAVR as an effective alternative to SAVR in appropriately selected low-risk populations, with long-term randomized follow-up demonstrating comparable survival and stroke outcomes and multiple studies demonstrating reduced postoperative morbidity [4,5,8,13]. Furthermore, observational mortality results suggest that decision-making should incorporate anatomy, comorbidity burden, durability expectations, and re-intervention feasibility. From a policy perspective, these findings reinforce guideline emphasis on patient evaluation and the need for longer-term registry surveillance to inform age-specific recommendations and durability monitoring.

Limitations

This systematic review is limited by heterogeneity in study design, population selection, valve generation, and follow-up duration. Furthermore, observational data are subject to residual confounding despite matching strategies [6-9]. Finally, the rapid evolution of TAVR technology means that long-term outcomes derived from earlier devices may not fully represent current performance.

Ethical Considerations and Future Research

The expansion of TAVR to low-risk populations introduces important ethical considerations. Clinicians must ensure informed patient decision-making by clearly communicating the benefits, risks, and uncertainties associated with both TAVR and SAVR, particularly regarding long-term valve durability and the potential need for future interventions. Equity in access is also critical, as TAVR may be less available or more costly in certain healthcare settings, raising concerns about fair distribution of care. Additionally, balancing short-term procedural benefits against uncertain long-term outcomes requires careful attention to the ethical principles of beneficence and non-maleficence.

Finally, future studies should focus on longer follow-up to better understand how TAVR and SAVR perform over time. Follow-up beyond 10-15 years will be important to evaluate valve durability, repeat procedures, and long-term survival. Research should also use more consistent definitions and reporting of outcomes. Finally, incorporating patient-centered outcomes such as functional status and quality of life will help guide treatment decisions and better reflect real-world priorities.

## Conclusions

TAVR and SAVR demonstrate similar long-term survival and stroke outcomes in randomized low-risk populations, while TAVR is associated with lower postoperative morbidity. Observational studies suggest potential differences in late outcomes among patients <65 and 60 years of age, emphasizing the importance of patient selection and long-term follow-up. As the use of TAVR continues to expand, treatment decisions should prioritize individualized management strategies that balance early procedural benefits with long-term patient outcomes.
